# An *Escherichia coli* strain for expression of the connexin45 carboxyl terminus attached to the 4th transmembrane domain

**DOI:** 10.3389/fphar.2013.00106

**Published:** 2013-08-23

**Authors:** Jennifer L. Kopanic, Mona Al-Mugotir, Sydney Zach, Srustidhar Das, Rosslyn Grosely, Paul L. Sorgen

**Affiliations:** Department of Biochemistry and Molecular Biology, University of Nebraska Medical CenterOmaha, NE, USA

**Keywords:** membrane protein expression, rare codons, connexins, NMR, minimal media

## Abstract

A major problem for structural characterization of membrane proteins, such as connexins, by nuclear magnetic resonance (NMR) occurs at the initial step of the process, the production of sufficient amounts of protein. This occurs because proteins must be expressed in minimal based media. Here, we describe an expression system for membrane proteins that significantly improves yield by addressing two common problems, cell toxicity caused by protein translation and codon bias between genomes. This work provides researchers with a cost-effective tool for NMR and other biophysical studies, to use when faced with little-to-no expression of eukaryotic membrane proteins in *Escherichia coli *expression systems.

## INTRODUCTION

Membrane proteins play a fundamental role in human disease and constitute a major portion of drug targets; lacking is sufficient structural and functional information compared to soluble proteins (Drew et al., 2003; Molina et al., 2008). The limitations can be traced to difficulties in expression, optimizing purification procedures, and reconstituting the proper fold in a lipid environment. Yield is a major problem because only a few membrane proteins are expressed in large-enough quantities to be collected from natural sources. Strategies developed to overcome this problem include engineering vectors to express membrane proteins in *S. cerevisiae* yeast, Sf9 insect, and *Escherichia coli* bacteria cell expression systems (Bernaudat et al., 2011).

*Escherichia coli* is a widely used host for the production of heterologous proteins due to its ability to grow rapidly at high density and in inexpensive substrates (Molina et al., 2008). The *E. coli* strain BL21 is extensively used for protein expression because it is deficient in lon and ompT proteases (Ratelade et al., 2009). The BL21(DE3) version carries a chromosomal copy of the T7 RNA polymerase gene under control of the lacUV5 promoter, suitable for protein production from target genes cloned into any T7 vector (e.g., pET) by induction with isopropyl-β-D-thiogalactopyranoside (IPTG; Studier and Moffatt, 1986; Unger et al., 1999). However, problems still arise because bacteria have difficulties folding membrane proteins and expression can be toxic (Miroux and Walker, 1996; Laible et al., 2004). Derivatives of BL21(DE3) called the Walker strains, C41(DE3) and C43(DE3), were therefore created with an enhanced ability to express otherwise toxic membrane proteins (Miroux and Walker, 1996). C41(DE3) was derived from the BL21(DE3) strain through natural selection to survive expression of the oxoglutarate-malate carrier protein from mitochondrial membranes. C41(DE3) has at least one uncharacterized mutation, enabling membrane protein expression into inclusion bodies without toxic effects (Miroux and Walker, 1996). Because expression of other membrane proteins were still toxic in the C41(DE3) strain, C43(DE3) was derived from C41(DE3) by selecting resistance to the F-ATPase b subunit gene. Thus, C43(DE3) can express a different set of toxic membrane proteins than C41(DE3).

Eukaryotic protein expression by *E. coli* is also strongly affected by codon bias. The genetic code contains 64 possible nucleotide combinations, which encode 20 amino acids and three codons that terminate translation. The frequencies with which different codons are used, which correlates with the amount of their corresponding tRNAs, vary between organisms (Gouy and Gautier, 1982). For example, eukaryotes commonly use the AGG codon for Arginine, which is rarely used in *E. coli* (Novy et al., 2001; Gustafsson et al., 2004). Expressing an eukaryotic gene with numerous rare codons in bacteria can impact expression through premature termination of translation, translational stalling, frame shifting, and mis-incorporation of amino acids (Kurland and Gallant, 1996). This problem can be solved by exchanging rare codons in the target gene for more frequently used codons in *E. coli* or by expressing the rare tRNAs. The latter has been implemented through creation of the BL21(DE3)-derived Rosetta (Novagen) and BL21(DE3)-CodonPlus (Agilent Technologies) strains. These strains contain a plasmid to express eukaryotic tRNAs rarely used in *E. coli*. For example, the pLysS plasmid within the Rosetta 2(DE3)pLysS strain carries tRNA genes that encode for seven rare codons, including AGG (Novy et al., 2001). Many studies have shown that protein expression is enhanced in these strains (Kane, 1995).

Every membrane protein is unique in the challenges needed to obtain a sample viable for structural studies. The increased number of possible methodologies at each step will help save researcher’s time and money, and more importantly may provide ideas for future improvements. Previous Cx43 studies from our laboratory identified that tethering of the CT domain to TM4 was necessary to elicit a change in secondary structure in response to factors known to regulate gap junction channels (Kellezi et al., 2008; Grosely et al., 2013). Therefore, our studies were extended to test the expression of other connexin carboxyl-terminal domains when attached to their 4th transmembrane domain (TM4-CxCT; Cx26, Cx32, Cx37, Cx40, Cx45, and Cx50). This is the first critical step toward structural characterization of their CT domains. These isoforms were chosen for investigated because of their known functional significance and involvement in human disease (for review, see Laird, 2010; Zoidl and Dermietzel, 2010). The protocol developed for TM4-Cx45CT expression will be described in detail, as an example of the TM4-CxCT domains. Cx45 is highlighted because of the unique expression requirements in comparison to the other isoforms. Cx45 is the first cardiac connexin expressed during embryonic development and plays an important role in propagating the action potential from the conduction system to the working myocardium (Severs et al., 2006; Palacios-Prado et al., 2010). Cx45 gap junction channels close when the membrane potential becomes negative, which has been suggested to prevent retrograde conduction from the myocardium to the conduction system (Palacios-Prado et al., 2010). Cx45 has limited expression in normal, working ventricular myocytes; however, in failing heart tissue, an up-regulation of Cx45 reduces the cell-to-cell coupling while promoting arrhythmogenesis, especially when superimposed on the down-regulation of Cx43 (Yamada et al., 2003; Betsuyaku et al., 2005).

Development of the methods described herein has resulted in protein yields that are at the levels necessary for biophysical characterization (e.g., circular dichroïsm (CD), isothermal calorimetry, etc.), including structural analysis by nuclear magnetic resonance (NMR). This methodology will be of general usage for other intrinsically ordered domains from membrane proteins.

## MATERIALS AND METHODS

### GENERATION OF THE TM4-CxCT CONSTRUCTS

**FIGURE 1:**
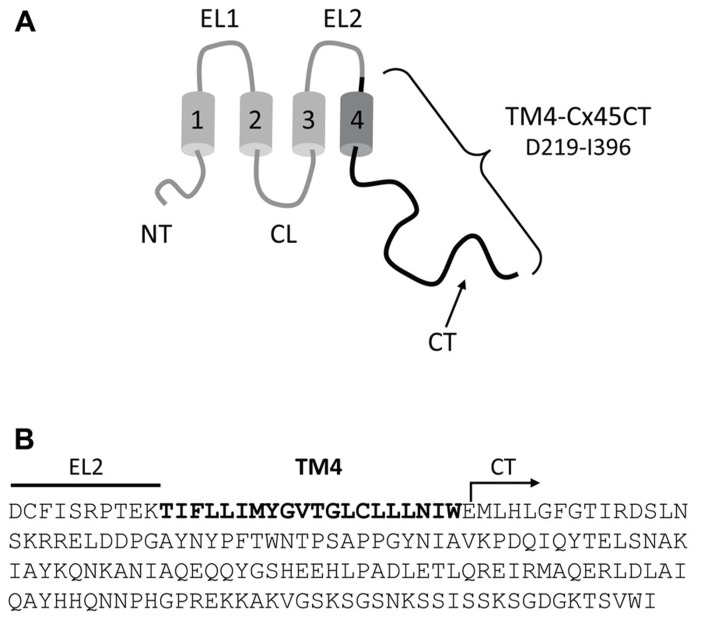
**Model of the TM4-Cx45CT construct. (A)** Schematic diagram of full length Cx45. The black coloring represents the TM4-Cx45CT construct. **(B)** The amino acid sequence of the TM4-Cx45CT (D219-I396) contains a portion of the EL2 (line) and the entire TM4 (bold) and CT (arrow) domains.

TM4-CxCT domains used in this study were from the Cx50, Cx45, Cx43, Cx40, Cx37, Cx32, and Cx26 isoforms. **Table 1** provides the amino acid sequence and species used for each TM4-CT domain to clone and ligate into the pET-14b expression vector (N-terminal 6× His-tag, ampicillin resistance; Novagen). Each construct includes 10 residues prior to their predicted TM4 domain (e.g., TM4-Cx45CT, **Figure 1**). All plasmid sequences were verified at the University of Nebraska Medical Center DNA Sequencing Core Facility.

**Table 1:** Conditions used to produce NMR samples for each TM4-CxCT construct.

Connexin isoform	Species	Optimal cell line	LB media^‡^	M63 minimal media^*^
TM4-Cx26CT (D179-V226)	*Homo sapiens*	C41	1.5	6
TM4-Cx32CT (D178-C283)	*Rattus norvegicus*	C41, C41Rt, R2	1.5	6
TM4-Cx37CT (D197-V333)	*Mus musculus*	C41, C41Rt	6	8
TM4-Cx40CT (N194-V356)	*Rattus norvegicus*	C41	10	12
TM4-Cx43CT (D197-I382)	*Rattus norvegicus*	C41	12	12
TM4-Cx45CT (D219-I396)	*Mus musculus*	C41Rt	10	8^*^
TM4-Cx50CT (D200-I440)	*Mus musculus*	C41, C41Rt, BL21	1	2

*Abbreviations used for *E. coli *competent cell stains: C41, C41(DE3); R2, Rosetta2(DE3)pLysSRARE2; C41Rt, C41(DE3)pLys; BL21, BL21(DE3).*

‡ Liters of bacterial culture required to produce a 300 μL sample at 1 mM concentration.

* ISOGRO required for TM4-Cx45CT expression in minimal media.

### PURIFICATION OF THE TM4-Cx45CT

Purification of the TM4-Cx45CT was based on the protocol developed for the TM4-Cx43CT domain (Kellezi et al., 2008). Cells were resuspended in 1× PBS buffer containing a bacterial protease inhibitor cocktail (250 μL/4 L cells; Sigma-Aldrich) and 1 mM β-mercaptoethanol. Cells were then lysed with an Emulsiflex-C3 (Avestin) for three passages at 15,000 psi. Cell debris was removed with centrifugation (4,000 rpm, 1 h) and a pellet containing the inclusion bodies was collected by centrifugation (18,000 rpm, 1 h). The pellet was resuspended in 50 mL Buffer A (6 M urea, 1× PBS, 20 mM imidazole, and 1 mM β-mercaptoethanol, 1% Triton X-100, pH 8.0) and rocked overnight at 4°C. The suspension was centrifuged again (18,500 rpm, 1 h), and the supernatant was loaded onto an ÄKTA FPLC using a HisTrap HP column (GE Healthcare). Protein elution was accomplished using a step gradient (4, 8, 10, 30, and 50%) of Buffer B (6 M urea, 1× PBS, 1 M imidazole, 1 mM β-mercaptoethanol, and 1% Triton X-100, pH 8.0). Fractions that contained the 22 kDa His-tagged TM4-Cx45CT protein (verified by SDS-PAGE and Western blot analyses) were pooled and dialyzed overnight at 4°C using a 10,000 MW cut off Slide-A-Lyzer dialysis cassette (Pierce) against buffer C [1 M urea, 1 mM dithiothreitol (DTT), 1 mM EDTA, and 1% Triton X-100]. The precipitate was collected and centrifuged (4,000 rpm, 5 min), washed twice with water then buffer D (20 mM MES buffer, 1 mM DTT, 1 mM EDTA, and 50 mM NaCl, pH 5.8). The washed precipitate was then solubilized in buffer E (20 mM MES, 1 mM DTT, 8% 1-palmitoyl-2-hydroxy-sn-glycero-3-[phospho-RAC-(1-glycerol)] (LPPG; Avanti Lipids), and 1 mM EDTA) and incubated at 42°C for 30 min. Buffer E was used for all TM4-Cx45CT experiments.

### WESTERN BLOT ANALYSIS

Protein samples were separated on 15% SDS-PAGE gels and transferred to a 0.45 μ polyvinylidene difluoride membrane (Millipore) equilibrated in transfer buffer (192 mM glycine, 25 mM Tris, 0.05% SDS, 10% methanol) using an electrophoretic transfer cell for 90 min at 100 V. After incubation with 5% non-fat milk in 1× PBS for 2.5 h, membranes were incubated with either mouse monoclonal anti-Cx45 (1:2,000, Cx45CR1 clone P3C9, Fred Hutchinson Cancer Center) or anti-His (1:2,000, His-Tag 27E9, Cell Signaling Technology, gift from Dr. Surinder Batra) for 16 h, 4°C. Membranes were washed four times at room temperature with washing buffer (0.1% Tween-20 in 1× PBS) for 10 min. Membranes were then incubated with goat anti-mouse IgG secondary antibody horseradish peroxidase conjugates (1:12,000, 12-349, Millipore) for 1 h, 25°C, then washed again. Bound antibodies were visualized using SuperSignal West Femto (Thermo Scientific).

### NUCLEAR MAGNETIC RESONANCE SPECTROSCOPY

All NMR data were acquired using a 600 MHz Varian INOVA NMR Spectrometer outfitted with a cryo-probe at the University of Nebraska Medical Center’s NMR Facility. NMR spectra were processed and phased using NMRPipe and NMRDraw (Delaglio et al., 1995) and analyzed using NMRView (Johnson, 2004). Gradient-enhanced two-dimensional ^15^N-HSQC experiments were acquired with 1,024 complex points in the direct dimension and 256 complex points in the indirect dimension (Kay et al., 1992). Sweep widths were 10,000 Hz in the ^1^H dimension and 2,430.6 Hz in the ^15^N dimension.

### CIRCULAR DICHROÄSM SPECTROSCOPY

Circular dichroïsm experiments were performed using a Jasco J-815 spectrophotometer fitted with a Peltier temperature control system. For each sample, five scans (wavelength range: 300–190 nm; response time: 1 s; scan rate: 50 nm/min; bandwidth 1.0 nm) were collected using a 0.01 cm quartz cell and processed using Spectra Analysis (Jasco). Each spectrum is shown as the mean residue ellipticity (MRE; deg cm^2^ dmol^-^^1^) as a function of wavelength and average of five scans. All spectra were corrected by subtracting the solvent spectrum. Protein concentrations were determined using a NanoDrop 1000 (Thermo Scientific) or Biospec 1601 UV-VIS spectrophotometer (Shimadzu) at 280 nm. Analyses of spectra were accomplished using the Provencher and Glöckner method with the SP175 reference set on the online program DichroWeb (Provencher and Glöckner, 1981; Whitmore and Wallace, 2004; Lees et al., 2006).

## RESULTS

### BACTERIAL STRAINS USED FOR PROTEIN EXPRESSION

**FIGURE 2:**
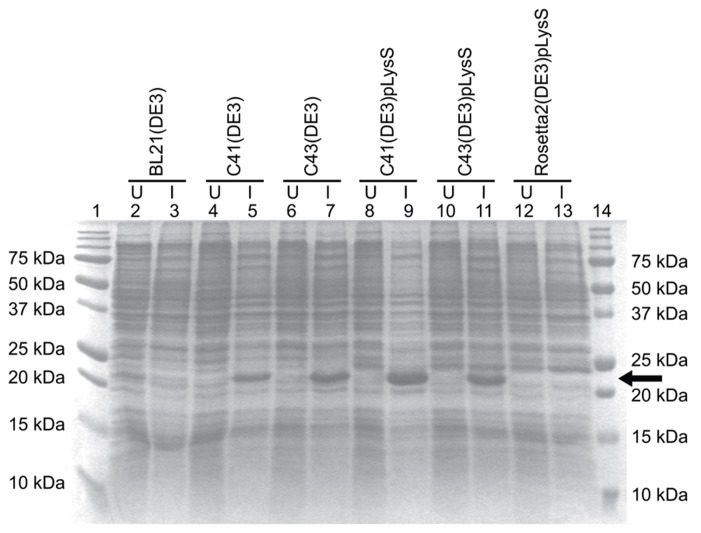
**TM4-Cx45CT expression profile obtained from different *E. coli* (DE3) strains with and without the pLysS plasmid in LB medium.** TM4-Cx45CT was expressed in the following *E. coli* cell strains: BL21(DE3) (lanes 2 and 3), C41(DE3) (lanes 4 and 5), C43(DE3) (lanes 6 and 7), C41(DE3)pLysS (lanes 8 and 9), C43(DE3)pLysS (lanes 10 and 11), and Rosetta 2(DE3)pLysS (lanes 12 and 13). Lanes 1 and 14 contain the Precision Plus Protein All Blue Standards molecular mass marker (Bio-Rad). Samples were collected just prior to (uninduced; U) and 4 h after IPTG induction (I), as noted. All lanes contain an equal amount of total protein for comparison. This was accomplished by pelleting 500 μL samples with an Abs_600__nm_ at 0.5 and resuspending the pellet in 30 μL of 6× SDS loading buffer. Five microliters of the samples were ran on a 15% SDS-PAGE gel and stained with Coomassie Blue. The TM4-Cx45CT has an expected molecular mass of ~22 kDa, indicated by the arrow.

The *E. coli* strains BL21(DE3), C41(DE3), C43(DE3), and Rosetta 2(DE3)pLysS (chloramphenicol resistance) were transformed with the TM4-Cx45CT plasmid and incubated in lysogeny broth (LB) medium at 37°C, 250 rpm. Rosetta 2(DE3)pLysS expresses seven rare codons (Arg, AGA, AGG, CGA, CGG; Ile, AUA; Pro, CCC, Leu, CUA) in comparison to the BL21-Codon Plus(DE3)-RIPL strain (contains the most amount of tRNA genes in the BL21-Codon Plus series), which contains only five tRNA genes (Arg, AGA, AGG; Ile, AUA; Pro, CCC, Leu, CUA). The TM4-Cx45CT contains five Arg rare codons, including two CGA, which the BL21-Codon Plus(DE3)-RIPL strain does not have the corresponding tRNA. Therefore, Rosetta 2(DE3)pLysS was chosen as the representative strain that expresses rare codons for this study. Protein expression was induced by the addition of 1.0 mM IPTG (final concentration; Bioexpress) at an optical density of 0.6 at 600 nm. The electrophoretic profile of total cellular proteins obtained 4 h after induction indicated that only C41(DE3) and C43(DE3) expressed TM4-Cx45CT (**Figure 2**, lanes 5 and 7, respectively). However, this protein yield is not optimal as significantly less protein per liter was produced then what was needed for the TM4-Cx43CT NMR structural studies (Kellezi et al., 2008). Upon examination of the TM4-Cx45CT gene sequence, 14 rare codons were identified; of these, eight (two in tandem) translate to the amino acid with the greatest codon bias, Arg (**Table 2**). Even though the Rosetta 2(DE3)pLysS strain was unable to express TM4-Cx45CT (Figure 2, lane 13), we hypothesized that expression would be enhanced by combining the pLysS plasmid with the C41(DE3) or C43(DE3) strains.

**Table 2:** Rare codon usage for each TM4-CxCT domain.

Connexin isoform	Number of rare codons^‡^					
	Arginine	Isoleucine	Leucine	Proline	Total	Tandem
TM4-Cx26CT (D179-V226)	0 AGG	0	1 CTA	2 CCC	4	No
	1 AGA					
	0 CGA					
TM4-Cx32CT (D178-C283)	0 AGG	1 ATA	0	3 CCC	5	No
	1 AGA					
	1 CGA					
TM4-Cx37CT (D197-V333)	1 AGG	1 ATA	0	8 CCC	15	No
	2 AGA					
	3 CGA					
TM4-Cx40CT (N194-V356)	2 AGG	0	1 CTA	4 CCC	8	No
	0 AGA					
	1 CGA					
TM4-Cx43CT (D197-I382)	1 AGG	0	0	2 CCC	7	No
	3 AGA					
	1 CGA					
TM4-Cx45CT (D219-I396)	2 AGG	1 ATA	3 CTA	4 CCC	14	Yes
	2 AGA					
	2 CGA					
TM4-Cx50CT (D200-I440)	5 AGG	2 ATA	1 CTA	3 CCC	12	No
	1 AGA					
	0 CGA					

‡ Number of rare codons was determined using the Rare Codon Calculator (.

### EXPRESSION OF TM4-Cx45CT WITH THE pLysS PLASMID

**FIGURE 3:**
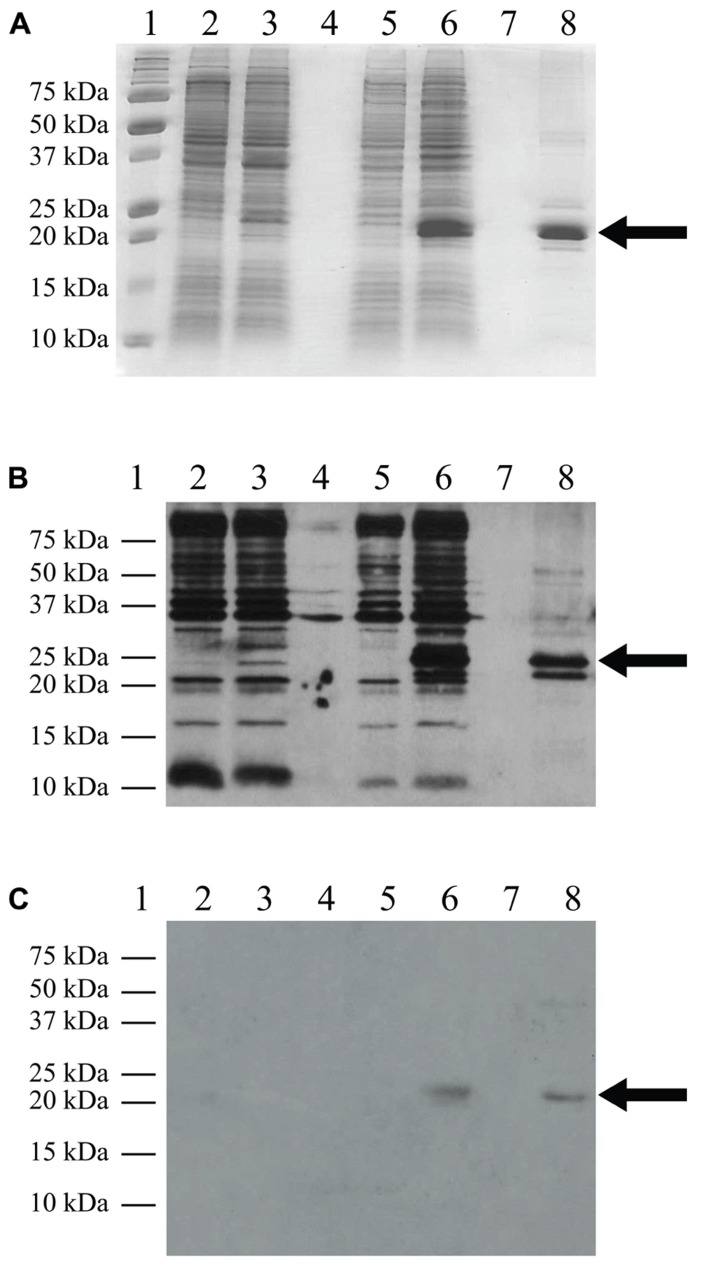
**Western blot analysis.** C41(DE3)pLysS transformed with the empty pET-14b vector (lanes 2 and 3) or the TM4-Cx45CT plasmid (lanes 5 and 6). Lane 8 contains the TM4-Cx45CT after purification. Lane 1 contains the Precision Plus Protein All Blue Standards molecular mass marker (Bio-Rad) and lanes 4 and 7 are blank. Samples were collected just prior to (lanes 2 and 5) and 4 h after IPTG induction (lanes 3 and 6). A total of 500 μL samples with an Abs_600__nm_ at 0.5 were pelleted and resuspended in 30 μL of 6× SDS loading buffer. Equal amounts of total protein (7 μL) were ran on a 15% SDS-PAGE gel. **(A)** Coomassie Blue stained gel is shown as a reference. Western blot analyses were performed using either **(B)** anti-His6, or **(C)** anti-Cx45 primary antibodies. The expected molecular mass of the TM4-Cx45CT is ~22 kDa, which is indicated by the arrow. Of note, in **(B)** lane 8, the anti-His6 primary antibody also reacted with a ~20 Da protein. Although not present in the **(A)** 15% SDS-PAGE gel or reactive with the **(C)** anti-Cx45 primary antibody, we speculate the doublet is caused by proteolysis of the TM4-Cx45CT.

The pLysS (also referred to as pLysSRARE2) plasmid was isolated from Rosetta 2(DE3)pLysS cells using the QIAprep Spin Miniprep Kit (Qiagen) and co-transformed with the TM4-Cx45CT plasmid into the C41(DE3) and C43(DE3) strains. The cells were grown and induced as described above. The electrophoretic profile of total cellular proteins obtained 4 h after induction showed that TM4-Cx45CT expression in C43(DE3) was similar with and without transformation of the pLysS plasmid (**Figure 2**, lanes 7 and 11, respectively). Conversely, TM4-Cx45CT expression was significantly increased in the C41(DE3)pLysS strain as compared to C41(DE3) alone (**Figure 2**, lanes 9 and 5, respectively). The expression was quantified by densitometry and revealed that the addition of the pLysS plasmid increased TM4-Cx45CT expression by 70% in C41(DE3). The expression of TM4-Cx45CT was confirmed by Western blot analyses using anti-His6 and anti-Cx45 antibodies (**Figure 3**).

**FIGURE 4:**
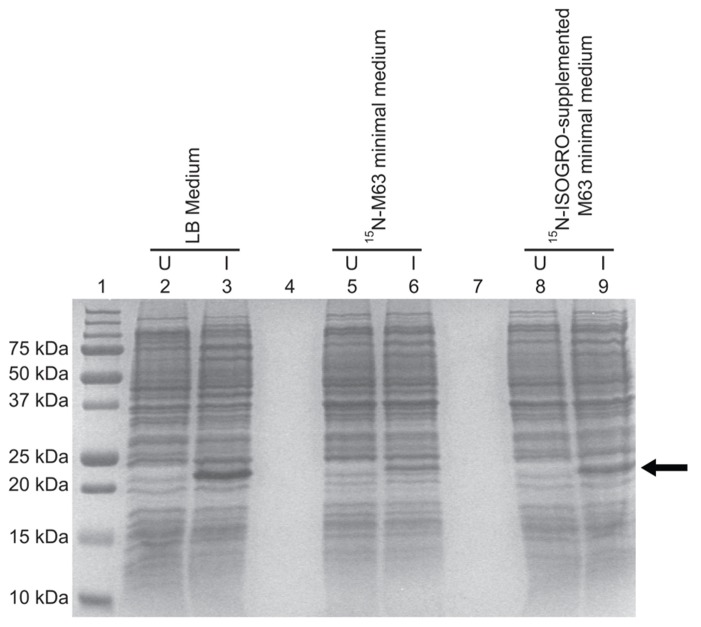
**Comparison of the TM4-Cx45CT expression profile obtained from *E. *coli strain C41(DE3) with the pLysS plasmid in LB and minimal media.** C41(DE3)pLysS transformed with the TM4-Cx45CT plasmid was grown in LB medium (lanes 2 and 3), ^15^N-labeled M63 minimal medium (lanes 5 and 6), and ^15^N-labeled M63 minimal medium supplemented with 1 g ^15^N-ISOGRO/L medium (lanes 8 and 9). Lane 1 contains the Precision Plus Protein All Blue Standards molecular mass marker (Bio-Rad) and lanes 4 and 7 are blank. Samples were collected just prior to (uninduced, U) and 4 h after IPTG induction (I), as noted. A total of 500 μL samples with an Abs_600__nm_ at 0.5 were pelleted and resuspended in 30 μL of 6× SDS loading buffer. Equal amounts of total protein (7 μL) were ran on a 15% SDS-PAGE gel and stained with Coomassie Blue. The TM4-Cx45CT has an expected molecular mass of ~22 kDa, indicated by the arrow.

Expression using the C41(DE3)pLysS strain was tested in isotopically labeled M63 minimal medium, which allows the control of nitrogen and carbon sources needed for NMR structural studies. The TM4-Cx45CT expression level decreased 84% in M63 as compared to LB (**Figure 4**, lanes 6 and 3, respectively). However, expression was restored to LB level when M63 was supplemented with ^15^N-ISOGRO (1 g/L, Isotec; **Figure 4**, lane 9). ISOGRO is an algal lysate-derived complex labeling medium that provides cells a metabolic boost that often decreases lag time, facilitates the attainment of growth saturation, and promotes recombinant protein production. ISOGRO helps cultures conserve cellular energy by limiting the requirement for *de novo* synthesis of cellular machinery and metabolic precursors; permitting more cellular resources to be direct toward recombinant protein expression. At this expression level, 8 L of growth is necessary to obtain a 1 mM at 300 μL volume (“gold standard” concentration and volume for NMR structural studies; **Table 1**). This is in contrast to the 54 or 180 L would be necessary in M63 without ^15^N-ISOGRO or without ^15^N-ISOGRO and the pLysS plasmid, respectively.

**FIGURE 5:**
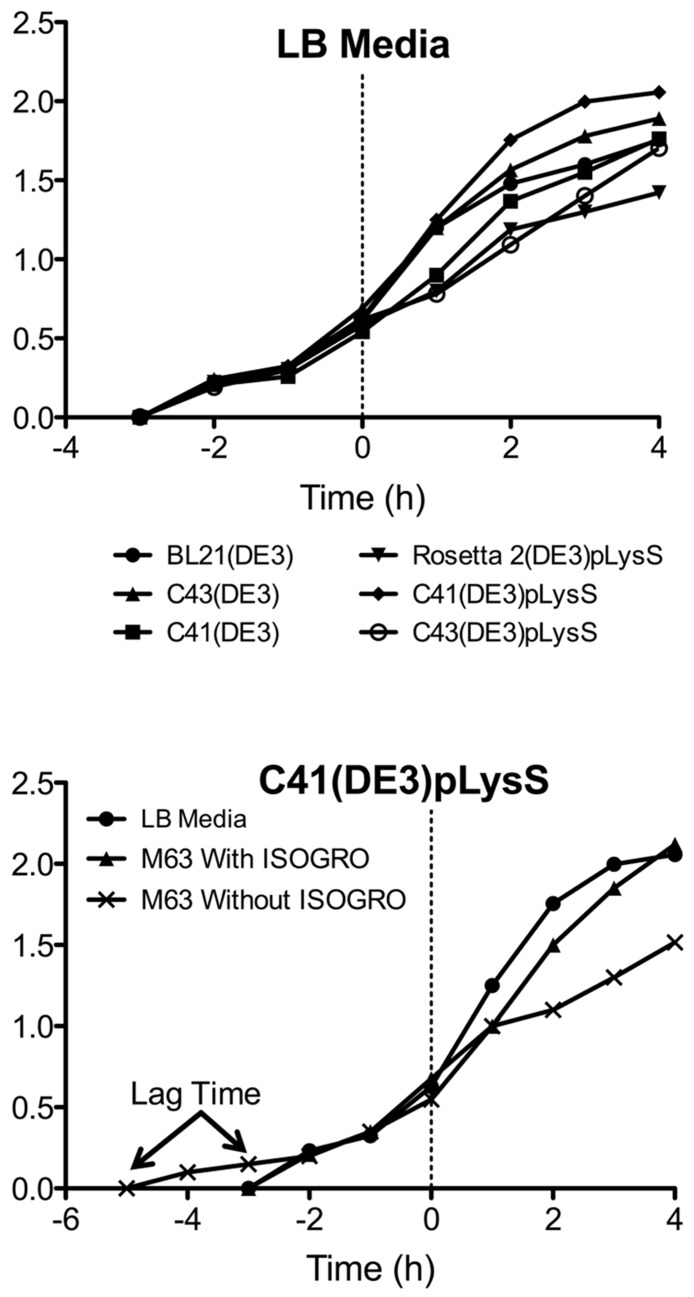
**Growth curves of the TM4-Cx45CT. (A)** Optical density of cultures in LB media were monitored at Abs_600__nm_ during the growth of TM4-Cx45CT in six different *E. coli* strains. **(B)** TM4-Cx45CT grown in the C41(DE3)pLysS stain using LB and M63 minimal media with or without ISOGRO. The dashed line at 0 h represents the induction with IPTG.

Another advantage of using the pLysS plasmid is the suppression of T7 RNA polymerase expression prior to induction with IPTG. The phenotypes observed from “leaky” expression (before IPTG induction) of a membrane protein that is toxic to *E. coli* are a slow growth rate, low cell density, and in some cases, cell death. The TM4-Cx45CT expression was not toxic to the *E. coli* strains tested as the growth rates and final cell densities were identical with and without the pLysS plasmid (**Figure 5**), indicating that the benefit gained from the pLysS plasmid was solely the tRNAs expression.

### EXPRESSION OF OTHER TM4-CxCT CONSTRUCTS

Outlined in **Table 1** are the results from the other six connexin isoforms. With the exception of the TM4-Cx45CT, all of the constructs were able to grow in C41(DE3) cells. When comparing the primary sequences of the connexin isoforms, they all contain rare codons (**Table 2**). However, TM4-Cx45CT is the only construct that contains rare codons in tandem, which is known to inhibit protein expression (Kim and Lee, 2006). In addition, the TM4-Cx32CT, TM4-Cx37CT, and TM4-Cx50CT constructs were also able to grow in the C41(DE3)pLysS strain. In general, the TM4-CxCT constructs produced more protein in LB media (e.g., for CD, ITC, etc.) as compared with minimal media (i.e., for NMR). The only differences were the TM4-Cx43CT isoform grew equally as well and the TM4-Cx45CT grew better in minimal media, which is caused by the addition of ^15^N-ISOGRO to the media.

### CHARACTERIZATION OF THE TM4-Cx45CT SECONDARY STRUCTURE

**FIGURE 6:**
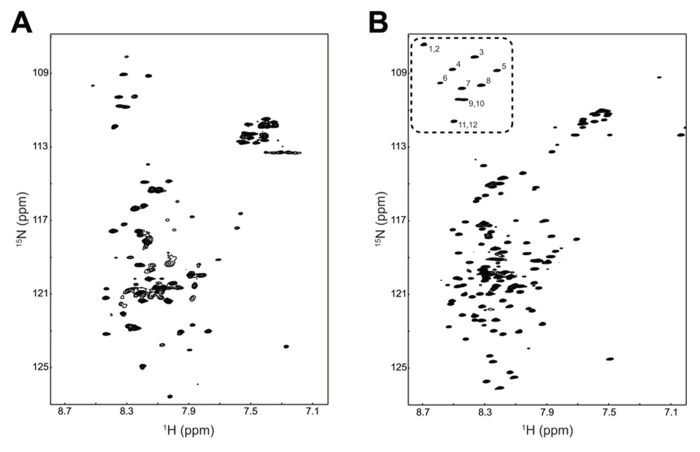
**Demonstrating the feasibility of solving the TM4-Cx45CT structure.** ^15^N-HSQC of the TM4-Cx45CT in 20 mM MES, 1 mM DTT, 1 mM EDTA, and 8% 1-palmitoyl-2-hydroxy-sn-glycero-3-[phospho-RAC-(1-glycerol)] detergent micelles **(A)** alone or **(B)** in the presence of 30% deuterated acetonitrile. Highlighted are the 12 Gly residues in the TM4-Cx45CT (dotted rectangle, numbered).

Combining a plasmid that expresses rare tRNAs with an *E. coli *strain selected to express toxic membrane proteins improved the yield of TM4-Cx45CT to levels that are cost-effective and now feasible for NMR structural studies. Using the expression protocol developed herein, the TM4-Cx45CT was purified and reconstituted into detergent micelles (LPPG) using techniques developed previously for the TM4-Cx43CT construct (Kellezi et al., 2008). The purity of the TM4-Cx45CT was verified by SDS-PAGE and Western blot analyses (**Figure 3**, Lane 8). Next, a ^15^N-HSQC spectrum was collected to evaluate the sample properties of the TM4-Cx45CT. The ^15^N-HSQC is a two-dimensional NMR experiment in which each amino acid except proline gives one signal, or chemical shift, that corresponds to the N–H amide group. **Figure 6A** shows the ^15^N-HSQC for TM4-Cx45CT collected in 20 mM MES, 1 mM DTT, 8% LPPG, and 1 mM EDTA. Unexpectedly, the spectra quality is poor, and only approximately 50% of the expected cross peaks are present. Previous studies identified that a soluble version of the Cx45CT (K265-I396) was in a dimer conformation that could be disrupted by acetonitrile (Kopanic and Sorgen, 2012). Therefore, a ^15^N-HSQC spectrum was collected in the presence of 30% acetonitrile (**Figure 6B**), which shows the total number (168) of expected amide cross peaks corresponding to the non-proline residues of the TM4-Cx45CT. Additionally, the number of Gly residues (12, circled and numbered) matches the number found in the primary sequence and indicates that the TM4-Cx45CT construct is in a single conformation. Altogether, this demonstrates the feasibility of solving the monomeric TM4-Cx45CT structure.

**FIGURE 7:**
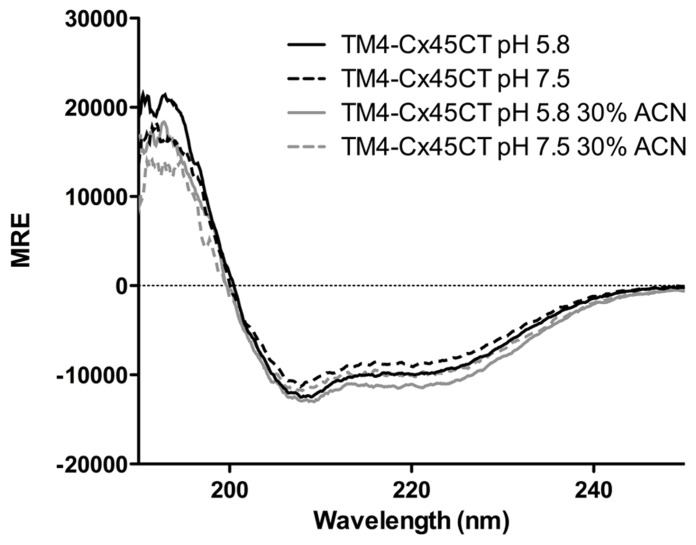
**Secondary structure of the TM4-Cx45CT.** Circular dichroïsm of the TM4-Cx45CT in 20 mM MES, 1 mM DTT, 1 mM EDTA, and 8% 1-palmitoyl-2-hydroxy-sn-glycero-3-[phospho-RAC-(1-glycerol)] detergent micelles alone (black) or in presence of 30% acetonitrile (gray) at pH 7.5 (solid lines) and 5.8 (dashed lines).

Circular dichroïsm was used to gain insight into the TM4-Cx45CT secondary structure before obtaining an atomic level structure. Intracellular acidification is a major consequence of tissue ischemia during a myocardial infarction, which leads to closure and degradation of gap junction channels and can be a substrate for malignant ventricular arrhythmias (Lau, 2005). Therefore, data were collected at either physiological (pH 7.5) or ischemic (pH 5.8) conditions (**Figure 7**). The TM4-Cx45CT (without acetonitrile) has a small increase in α-helical content under acidic conditions (pH 7.5, 25%; pH 5.8, 28%). This pH-effect is similar in the presence of 30% acetonitrile with a small increase in overall α-helical content (pH 7.5 28%; pH 5.8, 32%). The pH-induced increase in α-helical content for the TM4-Cx45CT (3–4%) is smaller than observed for the TM4-Cx43CT (16%; Kellezi et al., 2008; Grosely et al., 2012).

## DISCUSSION

Even though the soluble versions of connexin CT domains have proven to be useful for describing mechanisms involved in gap junction regulation, several results indicate that these constructs may not be optimal. For example, the cryo-electron microscopy structure of the Cx43 mutant (truncated at residue T263) suggested that the N-terminal region of the CT domain (S255-T263) contains α-helical structure (Unger et al., 1999). In contrast, the NMR data for the soluble Cx43CT (S255-I382) identified these same residues as weak resonances, suggesting an exchange between an unstructured and α-helical conformations (Sorgen et al., 2004). Additionally, not all of the expected NOEs were observed in the two dynamic α-helical regions of the soluble CT structure. The TM4-tethered Cx43CT protein (D219-I382) solubilized in detergent micelles offers a more native-like construct for structural studies (Kellezi et al., 2008). CD and NMR data indicated that the TM4-Cx43CT has more α-helical content than can be attributed to solely the addition of the TM4 domain to the soluble CT domain (Kellezi et al., 2008; Grosely et al., 2012). In addition, the TM4-Cx43CT is also structurally responsive to the changes in pH and phosphorylation, unlike the soluble Cx43CT, indicating that this construct is a better model for the investigation of structural-based mechanisms behind gap junction channel regulation (Kellezi et al., 2008; Grosely et al., 2012). Extending this study to other connexin isoforms, such as Cx45, will allow future structural studies to characterize mechanisms of gap junction regulation. The motivation behind this study is that a better understanding of the similarities and differences in structure between connexin CT domains when attached to the TM4 can be exploited to aid in the design of chemical modifiers.

## Conflict of Interest Statement

The authors declare that the research was conducted in the absence of any commercial or financial relationships that could be construed as a potential conflict of interest.
